# Psoriasis epidemiology screening tool (PEST) is useful for the detection of psoriatic arthritis in the Japanese population

**DOI:** 10.1038/s41598-021-95620-4

**Published:** 2021-08-09

**Authors:** Ayako Setoyama, Yu Sawada, Natsuko Saito-Sasaki, Shun Ohmori, Daisuke Omoto, Kayo Yamamoto, Haruna Yoshioka, Etsuko Okada, Motonobu Nakamura

**Affiliations:** Department of Dermatology, University of Occupational and Environmental Health, 1-1, Iseigaoka, Yahatanishi-Ku, Kitakyushu, Fukuoka 807-8555 Japan

**Keywords:** Health care, Medical research

## Abstract

Psoriasis is a chronic inflammatory skin disease that involves various systemic organs and tissues and is characterized by scaly erythematous skin. Among the different types of psoriasis, psoriatic arthritis (PsA) is frequently reported, and occasionally develops into severe arthritis leading to joint dysfunction. There are various tools, especially questionnaires, to identify the presence of PsA in European and American populations; however, little is known about the utility of these tools in the Asian population. In this study, we investigated the utility of a representative tool, the psoriasis epidemiology screening tool (PEST) questionnaire, to identify PsA among Japanese patients with psoriasis. A total of 143 patients with psoriasis were enrolled in this study. Among them, 29 patients were diagnosed with PsA. The frequency of PsA was significantly increased in patients with PEST scores > 3, with a sensitivity of 93.1% and a specificity of 78.9%. Among the questions in the PEST questionnaire, “Have you ever had a swollen joint?” showed the highest frequency to answer “Yes” among patients with PsA. Univariate and multivariate analyses revealed that high PEST scores (> 3) was an independent variable in PsA patients. Taken together, our study suggests that the PEST questionnaire is a useful tool to identify PsA among Japanese patients with psoriasis.

## Introduction

Psoriasis is a chronic inflammatory skin disease characterized by scaly erythematous skin with inflammation and it involves various systemic organs. Patients have conditions such as psoriatic arthritis (PsA), cardiovascular disease, and bone fracture^1–4^. PsA has a relatively high frequency of incidence in psoriasis patients, compared to other organ complications, and develops into severe arthritis, leading to the disability of arthritis function^5^. Thus, identifying the presence of PsA is important. However, the detection of PsA is not satisfactory in the current clinical scenario.

To address these problems, the psoriasis epidemiology screening tool (PEST) questionnaire was developed as a modified version of the PsA questionnaire^6^. Although it consists of five simple questions, the PEST questionnaire presents high-quality results with 92% sensitivity and 78% specificity^7^. Because there are differences in the clinical characteristics of each country, additional validation is required to clarify the actual impact on the clinical detection of PsA in various countries.

The incidence of inflammatory skin diseases differs among countries. While the prevalence of psoriasis ranges from 0.70 to 8.50% in Norway and the United states^8–10^, it is relatively low in Asian countries with prevalence of only around 0.23–0.61%^8,11^. In addition to the differences in the environment and the tradition of their daily lifestyle, the responsible genes show different expression between patients with psoriasis in the Asian countries and other countries^12–14^. Although the usefulness of the PEST questionnaire to detect PsA has been validated in some Asian countries^15,16^, it remains unclear whether it can be used for the Japanese population.

In this study, we show the impact of the PEST questionnaire on the Japanese population. Our study proposes that the PEST questionnaire might be a useful tool to detect PsA in the Japanese population.

## Materials and methods

### Patients

Patients diagnosed with psoriasis and PsA were confirmed by both dermatologists and rheumatologists based on the CASPAR criteria at the University of Occupational and Environmental Health from July 2017 to September 2020. The diagnosis of psoriasis was based on clinical features and histopathological findings proven by more than two independent dermatologists. The psoriasis patients in our study were classified into three subtypes: psoriasis vulgaris, pustular psoriasis, and erythrodermic psoriasis^17^. Dermatologists initially examined whether these patients with psoriasis had clinical symptoms of peripheral arthritis in the extremities namely, enthesopathy, axial spondyloarthritis, and swelling of the fingers and toes. After the initial examination, further investigation was performed in cases suspected of PsA to confirm the diagnosis. The diagnosis of PsA was confirmed in psoriasis patients at the first visit and during follow-up.

### Clinical evaluations

The patients were classified into two groups according to age: 60 years or older, and younger than 60 years. Past history was also classified according to the presence of diabetes mellitus or hypertension. Uric acid and calcium levels were classified into two groups based on a standard index, which were defined as serum uric acid and calcium levels more than 7 mg/L and 10 mg/L, respectively. We categorized psoriasis patients into four different types: psoriasis vulgaris, psoriatic erythroderma, pustular psoriasis, and PsA.

### PEST questionnaire evaluation

PEST scoring was performed as previously described^6^. There are five questions as follows: (1) Have you ever had a swollen joint (or joint)?, (2) Has a doctor ever told you that you had arthritis?, (3) Do your fingernails or toenails have holes or pits?, (4) Have you experienced pain in your heel?, (5) Have you had a finger or toe that was completely swollen and painful for no apparent reason? We evaluated the total PEST score as the sum of “Yes” answers. The PEST questionnaire was administered during the first visit in the observation period. The diagnosis of PsA was made at that time, and at the follow-up duration of the observation period.

### Statistical analyses

To examine the comparisons of the factors and pairs of groups, Fisher’s exact test, chi-square test, or Student’s t-test was performed. The sensitivity and specificity were also calculated. The sensitivity and specificity were calculated as the proportion of positive and negative PsA diagnoses, respectively. Logistic regression analysis was conducted to exclude the influence of the variable factors. All statistical analyses were performed using JMP13.1 (SAS Institute Inc., Tokyo). Statistical significance was set at *p* < 0.05.

### Study approval

Our observational retrospective study using existing data was approved by the institutional review board of the University of Occupational and Environmental Health and was conducted in accordance with the Declaration of Helsinki. Informed consent was obtained from the patients based on the approval of the ethical committee of the University of Occupational and Environmental Health.

## Results

### The clinical characteristics of PsA

A total of 143 patients with psoriasis were enrolled in this study, and the total number of PsA and non-PsA patients were 29 and 114, respectively. The frequency of psoriasis itself varies across countries and areas, and the characteristics of the population differ depending on the country and its facilities. Therefore, to gain a better understanding of the characteristics of PsA patients in our study, we first analyzed the characteristics of PsA in our population. Among these patients, 29 were diagnosed with PsA, and the prevalence in this study was 20.7%. The distribution of arthritis is presented in Table 1. Finger and knee were the most common sites of arthritis in 52.0% of PsA patients, followed by the Achilles tendon (38%), sole (35%), and wrist (33%). On the other hand, buttocks (6%) and jaw (2%) had a lower frequency of arthritis among patients with PsA.

**Table 1:** The arthritis distribution.

The distribution of arthritis	Percentage (%)
Neck	12
Jaw	2
Shoulder	31
Elbow	21
Wrist	33
Finger	52
Chest	10
Back	12
Waist	23
Buttocks	6
Knee	52
Toe	16
Achilles tendon	38
Sole	35

There is some overlapping in the distribution of arthritis.

### The clinical differences between PsA and non-PsA psoriasis

We examined the detailed differences in clinical characteristics between PsA and non-PsA psoriasis patients. Table 2 summarizes the clinical characteristics of the patients in our study. Among the various variables in clinical characteristics, there were significant differences in the frequency of age and a high frequency of younger age was observed in PsA patients. We conducted blood tests for serum uric acid and calcium levels in 104 and 111 patients, respectively. We could not find a significant difference in uric acid levels between patients with psoriasis and PsA. In addition, there were no significant differences in sex, history of diabetes mellitus or hypertension, or serum calcium levels between PsA and non-PsA psoriasis patients.

**Table 2:** Clinical characteristics of psoriasis and psoriasis arthritis.

	Psoriasis	PsA	Odds ratio	*P*-value
Total number	114	29		
Sex				
Male	75	16	1.56	0.289
Female	39	13		
Age				
< 60	52 (45.6%)	21 (72.4%)	0.32	0.009
> 60	62 (54.4%)	8 (27.6%)		
Past history				
DM	19 (17.1%)	3 (10.7%)	0.58	0.407
HT	36 (32.4%)	9 (32.1%)	0.99	0.977
Laboratory examination				
UA ≧7	25 (30.1%)	4 (19.1%)	0.546	0.312
< 7	58 (69.9%)	17 (80.9%)		
Ca ≧10	14 (16.1%)	1 (4.2%)	0.227	0.130
< 10	73 (83.9%)	23 (95.8%)		
Pest score				
2 <	90 (78.9%)	2 (6.9%)	50.63	< 0.0001
3 >	24 (21.1%)	27 (93.1%)		

*P*-value was evaluated by Fisher exact test or Chi-square test.

Next, we investigated the involvement of the PEST questionnaire in our population. A total PEST score of more than 3 was the threshold to identify PsA patients with a significant difference (Table 2), as previously described^18^, and the sensitivity and specificity were 93.1% (27/29 patients with PsA with a PEST score > 3) and 78.9% (90/114 patients with non-PsA with a PEST score less than 2), respectively (Table 2). Based on these findings, we used this threshold as a high PEST score for further investigation.

### The tendency of results in the PEST questionnaire

**Figure 1:**
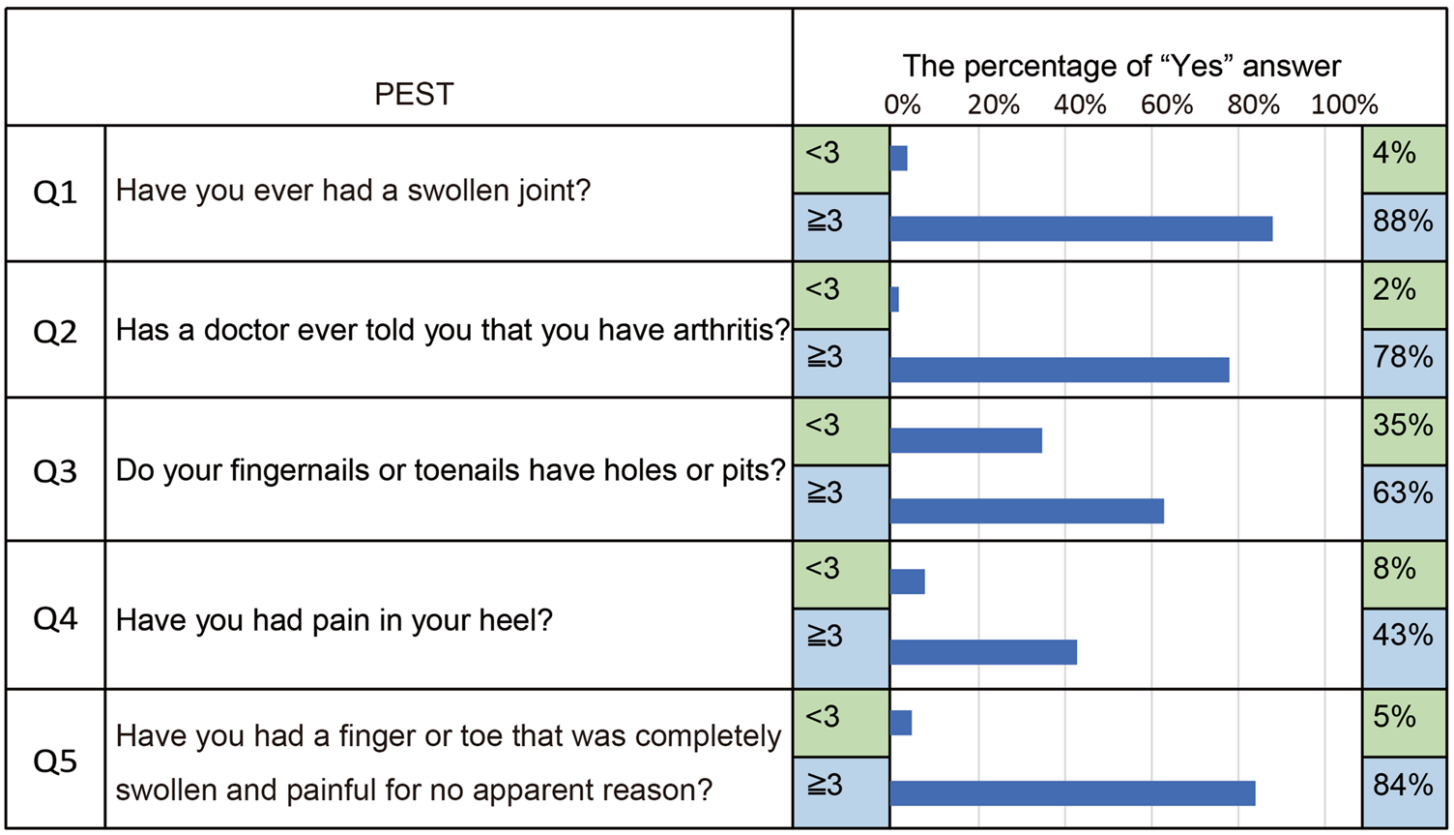
The results of PEST questionnaire. The different distribution of 5 questions in PEST questionnaire was analyzed between PEST score high and low patients.

The PEST questionnaire consists of five questions, and it has been reported that there is a tendency to answer “Yes” in the question^6,18^. Nevertheless, the tendency of the Japanese population to select among these five options remains unclear. To get a better understanding of the characteristics of a high PEST score in our study, we analyzed the detailed contexts of the questionnaire to answer “Yes.” The highest frequency to answer “Yes” was from the question “Have you ever had a swollen joint?” in which was seen in 88% of patients with a high PEST score (Fig. 1). On the contrary, the highest frequency of answering “Yes” in patients with low PEST score was “Do your fingernails or toenails have holes or pits?”, which was seen in 35% of patients. The lowest frequency to answer “Yes” was “Have you had pain in your heel?” seen in 43% in patients with a high PEST score.

### The clinical differences between patients with high and low

To explore the differences in clinical characteristics between patients with high and low PEST scores, we summarized the clinical characteristics of these patients in our study, as shown in Table 3. There were 92 and 51 patients with high and low PEST scores, respectively. In this analysis, we noticed that the age of ≥ 60 years was significantly increased in patients with high PEST scores. In addition, serum calcium levels were also significantly increased in patients with high PEST scores. On the contrary, there were no significant differences in sex, history of diabetes mellitus or hypertension, or serum uric acid level.

**Table 3:** Clinical characteristics classified into PEST score.

	PEST < 3	PEST≧3	Odds ratio	*P*-value
Total number	92	51		
Sex				
Male	63	28	1.78	0.106
Female	29	23		
Age				
< 60	40	33	0.420	0.015
≧ 60	52	18		
Past history				
None	50	26		
DM	14	8	1.06	0.905
HT	28	17	1.18	0.666
Laboratory examination				
UA≧7	21 (31.8%)	8 (21.1%)	0.571	0.238
< 7	45 (68.2%)	30 (78.9%)		
Ca ≧10	13 (19.1%)	2 (4.65%)	0.206	0.030
< 10	55 (80.9%)	41 (95.4%)		

*P*-value was evaluated by Fisher exact test or Chi-square test.

The characteristic tendency of the distribution of skin eruptions in PsA patients has been reported^19^. To clarify the difference in our population based on dermatological aspects, we next examined the difference in the distribution of skin eruptions between patients with high and low PEST scores. Unexpectedly, our population exhibited no difference in the frequency of nail psoriasis and skin eruptions around the nail between patients with PsA and those without PsA (Table 4).

**Table 4:** The skin distribution based on PEST score.

The distribution of skin eruption	Psoriasis	
	Non-PsA	PsA
Head	55 (48%)	14 (48%)
Upper extremities	79 (69%)	23 (79%)
Trunk	71 (62%)	19 (66%)
Lower extremities	97 (85%)	22 (75%)
Skin eruption around nail and/or nail itself	50 (44%)	14 (48%)

There is some overlapping in the distribution of arthritis.

### A high PEST score was an independent variable in PsA patients

It is known that various clinical characteristics are involved in PsA. Although we found that the frequency of PsA was significantly increased in patients with a high PEST score, it is necessary to clarify whether the high PEST score is an independent variable in PsA patients. In addition, we also noticed that age of ≥ 60 years was significantly increased in patients with high PEST scores. Age is not a common clinical variable in PsA patients; therefore, we could not completely deny the possibility that these variables might contribute to the high prevalence of PsA in patients with high PEST scores. Consistent with our results, univariate analysis revealed a significant difference in the PEST score and age (Table 5). To solve this issue, we conducted a logistic regression multivariate analysis of our population to exclude the influence of other clinical parameters on the impact of the PEST questionnaire results. In this logistic regression analysis, we excluded the variables around the nail and nail eruption, because there were no significant differences in the frequency (Table 4) and univariate analysis (Supplementary Table 1), in addition to including this factor in the PEST questionnaire. Among these clinical variables, patients with high PEST scores showed a significant difference (odds ratio [OR] 2.44, *P* < 0.01) (Table 5).

**Table 5:** Univariate and multivariate analysis for clinical variables.

Clinical variables	Univariate			Multivariate		
	OR	95%CI	*P* value	OR	95%CI	*P* value
Age	0.95	0.92–0.99	0.01	0.96	0.91–1.01	0.145
Sex	0.73	0.27–2.04	0.553	0.83	0.22–3.14	0.783
DM	0.77	0.20–3-04	0.716	0.81	0.13–4.89	0.816
HT	1.59	0.57–4.42	0.372	2.23	0.45–10.9	0.317
UA	0.95	0.72–1.26	0.747	0.92	0.32–1.08	0.661
Ca	1.40	0.53–3.72	0.495	1.57	0.42–5.88	0.476
PASI	1.03	0.97–1.10	0.307	0.99	0.92–1.01	0.981
PEST	2.71	1.68–4.37	< 0.0001	2.44	1.50–3.97	0.0003

## Discussion

This study revealed the advantages of PEST in patients with psoriasis in the Japanese population. In our study, the total PEST score was significantly increased in PsA patients with 93.1% sensitivity and 78.9% specificity. Several previous studies identified that the PEST questionnaire showed high-quality results with 84% sensitivity and 75% specificity among psoriatic patients^20^. On the contrary, another study showed a low specificity of 45% among all patients with psoriasis patients^21^. This is reasonable according to the background in which these reflect the different circumstances and prevalence in each country. Our study showed a relatively high sensitivity and specificity in the PEST questionnaire. The reason remains unclear; however, this might reflect the characteristics of the Japanese population, and the PEST questionnaire which is focused on subjective symptoms. The Japanese population prefers to select the middle answer, namely “Yes and No answer,” and tend to have an honest character and evaluates moderation as a positive virtue^22^. These tendencies have also been observed in other East Asian populations^23^. Furthermore, the Japanese prefer not to select positive responses in an extreme questionnaire style^23^, possibly leading to a low false-positive ratio. Therefore, these features might contribute to the increasing quality of the PEST questionnaire in the Japanese population, suggesting that this questionnaire might be eligible for the Asian population to identify PsA among psoriasis patients.

The prevalence of PsA in Asian countries patients are still lower than that in non-Asian countries^8,11^. However, daily lifestyle crucially influences the development of psoriasis, and Japanese lifestyle is shifting to European and American styles. As a result, the frequency of obesity has gradually increased in the Japanese population. Daily lifestyle-related obesity is a representative variable between Asian and non-Asian populations. For instance, under obesity conditions, IL-17-dominant skin conditions are observed even in the steady state, and the abundance of IL-17-mediated inflammatory response is observed in obese mice by using imiquimod-induced psoriatic skin inflammation^24^. Therefore, it is assumed that the prevalence of psoriasis and PsA will increase with changes in daily style.

Because this was a single-institution study and our institution is the therapeutic center in the area, the PsA population in our study might have a higher prevalence compared to the general dermatology clinics in Japanese patients with psoriasis. Hence, we could not conclude the actual prevalence of PsA among patients with psoriasis in the Japanese population. To clarify the precise prevalence in the Japanese PsA population, a large investigation will be required in the future.

Previous studies have already shown a high frequency of skin eruptions around the nail or nail itself in PsA patients. The nail and enthesis are closely linked with the DIP joint tendon^25^. Therefore, PsA-associated inflammation of the DIP joint easily spreads into the nail matrix, which is reflected as a skin eruption around the nails and nail itself^25,26^. Unfortunately, this study could not clearly show the importance of the nail itself and/or skin eruption around the nail in PsA patients, possibly due to the difference in the background of our psoriasis patient population. However, the PEST questionnaire might still be useful for identifying patients with PsA, even though they exhibit less clinical findings of nail and around nail eruption.

As variables affecting the pathogenesis of psoriasis, hyperuricemia is known as an independent variable factor for PsA^27^, although our study did not find a significant contribution of hyperuricemia in PsA patients. On the basis of pathogenesis, the IL-23/IL-17 axis is one of the main contributors to the development of psoriasis, and this is consistent with the beneficial impact of biologics targeting this pathway^28^. Uric acid contributes to the pathogenesis of psoriasis by initiating the NALP3 inflammasome, which drives IL-1β-mediated IL-17-committed T cell induction^29–31^. Thus, hyperuricemia may cultivate Th17-dominant circumstances in the skin, resulting in hyperreactive cutaneous immunity to IL-23-mediated inflammatory responses. Furthermore, this axis promotes damage-associated molecular pattern recognition and serine proteinase production by infiltrated neutrophils, leading to further production of IL-1β from pro IL-1β^32,33^. Uric acid has an affinity to deposit into the joint and Achilles tendon as a crystal form^34,35^. Therefore, it is assumed that these cytokines, loop-triggered by hyperuricemia, contribute to the development of PsA. Patients with high PEST scores showed a significantly high frequency of hypercalcemia; however, our univariate and multivariate analyses did not show an association between hyperuricemia and the results of the PEST questionnaire. Therefore, it depends on the pre-existence of treatment for hyperuricemia. In addition, age in our PsA population might have affected the results. The causes of hyperuricemia are classified into three types according to a recent classification: renal overload, renal underexcretion, and combined type^34^. A relatively younger age in our population might have preserved their renal function less to cause underexcretion of uric acid from the internal body. Therefore, our PsA population did not reflect the importance of hyperuricemia in PsA. Although this PEST questionnaire could not become a complete alternative examination for hyperuricemia in PsA patients, it is helpful for dermatologists to use as an initial investigation for PsA patients. From a cost–benefit perspective, the PEST questionnaire might become an easy and low-cost examination in dermatology clinics.

This study has some limitations. This study was conducted at a single institution, and the number of patients included in the study was limited. In addition, only Japanese patients were enrolled in the study. Further exploration of the utility of the PEST questionnaire is needed in other Asian population.

In conclusion, we showed that the PEST questionnaire might be useful for the Japanese population. Because the prevalence of psoriasis and PsA in the Japanese population is significantly lower than that in European countries and America, multi-institution studies with larger numbers of patients are desirable to explore the actual impact of PEST in psoriasis patients in the Japanese population. Although this is a single-institution study, our results suggest a possible higher prevalence of PsA than previously thought.

## Supplementary Information

Supplementary Information.
