# Supplementary material for: Psoriasis epidemiology screening tool (PEST) is useful for the detection of psoriatic arthritis in the Japanese population
Source: Sci Rep. 2021 Aug 9;11:16146. doi: 10.1038/s41598-021-95620-4 (PMC8352892; doi:10.1038/s41598-021-95620-4)
Supplement: Supplementary file 1 — Supplementary Information. [file 41598_2021_95620_MOESM1_ESM.docx]

**Psoriasis Epidemiology Screening Tool (PEST) is useful for the detection of psoriasis arthritis in the Japanese population**

Ayako Setoyama^1*^, Yu Sawada^1*^, Natsuko Saito-Sasaki^1^, Shun Ohmori^1^, Daisuke Omoto^1^,

Kayo Yamamoto^1^, Haruna Yoshioka^1^, Etsuko Okada^1^, Motonobu Nakamura^1^

1. Department of Dermatology, University of Occupational and Environmental Health, Kitakyushu, Japan

*Corresponding author: Ayako Setoyama, MD., and Yu Sawada, MD., PhD.

e-mail: [ayako-s@med.uoeh-u.ac.jp](mailto:ayako-s@med.uoeh-u.ac.jp) (AS) and [long-ago@med.uoeh-u.ac.jp](mailto:long-ago@med.uoeh-u.ac.jp) (YS)

University of Occupational and Environmental Health.

1-1, Iseigaoka, Yahatanishi-Ku, Kitakyushu, Fukuoka, 807-8555, Japan

| Supplementary Table 1. Univariate analysis for skin eruption around nail and/or nail itself | | | |
| --- | --- | --- | --- |
| Clinical variable | Univariate | | |
|  | OR | 95%CI | P value |
| skin eruption around nail and/or nail itself | 1.19 | 0.53-2.70 | 0.670 |
|  | | | |
